# Editorial: Systems Biology of Transcription Regulation

**DOI:** 10.3389/fgene.2016.00124

**Published:** 2016-07-06

**Authors:** Ekaterina Shelest, Edgar Wingender

**Affiliations:** ^1^Leibniz Institute for Natural Product Research and Infection Biology, Hans-Knoell InstituteJena, Germany; ^2^Institute of Bioinformatics, University Medical Center GoettingenGoettingen, Germany

**Keywords:** systems biology, transcription regulation, regulatory networks, modeling

Systems biology (SB) is a holistic approach, an attempt to view a living system in its integrity. A system is thus considered as more than just a sum of its parts; interactions bring their flavor. Transcription regulation is in a way ideal for application of systems biology approaches, because it is complex and because it is a regulatory system. The latter puts it right in the middle of SB efforts, because regulation is central to any system: without regulation a system loses connections, its “systemic” property. Focusing on SB of transcriptional regulation, as we do in this Research Topic, is not stepping back into a reductionist approach. The complete signature of gene activities, their control, and consequences rather represents the status of a living system, for instance a single cell, in a comprehensive way. Here, we are in a good position to investigate the properties and patterns of regulatory circuits on different levels, from transcription regulation networks (TRNs) and signaling pathways to intercellular crosstalk, development, and further to physiological function on tissue and organism level—to that extent in which it depends on gene expression and its regulation.

That is more or less a perspective. Systems biology of transcription regulation, as any other systems biology, is not yet a field with a well-established set of standard methods. It is also not a field with well-defined borders and unambiguously understood content. On the one hand, the subject is too complex and simultaneously too broad, which opens a wide field of activity. On the other hand, regulation of transcription is since long in the focus of intensive research and understanding of some (usually quite narrow) parts of it is very much advanced. There is also a historical bias toward some “favorite” processes, model organisms, where we can find examples of amazing advances; however, for other, not yet well investigated processes we are often just at the stage of collecting “bricks” from which the future building of our understanding will be constructed.

This status of the SB of transcription regulation is reflected by the collection of articles in this issue. We can see the variety of views, methods, applications, and questions raised and answered: from application of state-of-the-art methods to a particular object (e.g., Wlochowitz et al.) to development of novel methods (Wachter and Beissbarth; Martignetti et al.), from discussions of critical methodological and technical issues (e.g., Madrigal) to detailed analysis of robustness mechanisms (Payne and Wagner), from first descriptions of pathways in a non-model plant (Iaria et al.) to advanced SB in well-established models (e.g., Ben-Tabou de-Leon, etc.). Let us briefly go through this collection.

For transcription regulation, at least in the part considering transcription factors (TFs), TF binding sites (TFBSs) form the basis of the pyramid. Boeva in her review leads us through the forest of existing tools for prediction of motifs and TFBSs, demonstrating in the end how application of these methods can improve the accuracy of peak-calling in CHIPSeq. TFBSs are also in the focus of the investigation of heart development regulation (Zeidler et al.). The findings suggest that TF interactions are stage-specific and support the hourglass model of heart development. Wlochowitz et al. apply the state-of-the-art tools, such as Trinity (Grabherr et al., 2011) and geneXplain (http://genexplain-platform.com/bioumlweb/), to find differences between two cancer cell lines in terms of master TFs and signaling pathways. Analyzing gene regulatory networks (GRNs) and pathway interplays, the authors come to the explanation of the invasive potential of different cancer. Transcriptome analysis is also central for the papers of Iaria et al. and Smita et al. In the former, gene expression was monitored during maturation of fruits in two olive cultivars, followed by comparative analysis and reconstruction of metabolic pathways involved in olive drupe development. This is a nice example of tissue-specific functional genomics in a non-model plant species. Smita et al. used “top-down” and “guide-gene” approaches to study transcriptome-based GRN of MYB TFs in rice. The observations of differential regulation of all 233 rice MYBs in GEO-derived microarray data along with the phylogenetic analysis demonstrated that phylogenetically close pairs of MYB TFs are involved in highly similar regulatory processes.

Bringing together different data layers is a typical SB challenge. In our Topic, we have two papers suggesting interesting approaches to it. Wachter and Beissbarth draw our attention to the fact that a lot of cellular signaling information is encoded in signaling dynamics. To take this into account, the authors suggest a novel pathway-based method for the analysis of coupled omics time-series data through inferring consensus profiles and time profile clusters. Another approach suggested by Offermann et al. is based on dynamic Boolean models inferred from time-resolved transcriptomes, protein, and phenotypic data. The models can be further optimized by fitting to experimental data and finally can describe temporal resolution of network events (regulation–transcription–feedback). Interestingly, in both papers the methods were applied to describe the same pathway, epidermal growth factor (EGF) signaling. Some new promising interactions were suggested by the first method. In the second application, EGF was confronted with NGF signaling with a very interesting outcome, suggesting that positive transcriptional feedback induces bistability in the switch between differentiation and proliferation, moreover, differentiation uses three redundant pathways.

A less typical problem is tackled by Martignetti et al.: how to estimate activity of genes based on expression data, for instance the activity of a TF from expression of its target genes? For that, the authors developed a software ROMA for quantification of the activity of gene sets with coordinated expression. Application examples demonstrate that the activity of a signaling pathway is better reflected by the set of regulated genes than by any of these genes taken individually, which is an important message for future SB applications.

The paper of Lizio et al. introduces experimental strategies to build cell-type specific TRNs. The authors use complementary approaches (CHIPseq, KD-CAGE) to identify genome-wide targets of genes of interest and warn about the problems that may arise by the usage of CHIPseq alone. This critical view is very important. Another kind of concern is expressed in the opinion paper of Madrigal, who raises a discussion of such serious issue as sequence-specific bias in chromatin assembly experiments. Indeed, this issue can be easily overlooked, and it is essential to be aware of the dangers of sequence (or any other) biases when designing an experiment or treating the results. Madrigal describes the types of bias in different analyses and the adequacy of current benchmarks.

The problem of reproducibility of individual analyses is raised by Berto et al. To extract the most confident and biologically relevant information, the authors developed a method for integration of independently derived networks into a consensus network. This approach was applied to such complex and highly variable systems as cognitive disorders.

Understanding of such properties as robustness can be only addressed from systemic perspective, making it central topic of several presented here papers. Payne and Wagner in their comprehensive review analyze the mechanisms of mutational robustness, discussing its causes and consequences. Another type of robustness—temporal control of developmental GRNs—is discussed by Ben-Tabou de-Leon. Analysis of network motifs helps us to understand how the network architecture supports the timely activation of regulatory and differentiation genes. Rigid motif combinations, such as a triple positive feedback loop conserved through bilateral, explain the robustness of the system, and suggest that this “approach” can be used in other systems as well.

Altogether, this comprehensive collection of articles provides a nice overview of the present status of SB of transcription regulation, demonstrating the advances in different areas achieved through the application of SB approaches.

## Author contributions

ES and EW have read all Research Topic articles, ES drafted the review, both authors wrote the paper and approved it for publication.

### Conflict of interest statement

The authors declare that the research was conducted in the absence of any commercial or financial relationships that could be construed as a potential conflict of interest.
